# Genetically heterogeneous mice exhibit a female survival advantage that is age‐ and site‐specific: Results from a large multi‐site study

**DOI:** 10.1111/acel.12905

**Published:** 2019-02-23

**Authors:** Catherine J. Cheng, Jonathan A. L. Gelfond, Randy Strong, James F. Nelson

**Affiliations:** ^1^ Department of Cell Systems & Anatomy UT Health San Antonio San Antonio Texas; ^2^ Barshop Institute for Longevity and Aging Studies, UT Health San Antonio San Antonio Texas; ^3^ Department of Epidemiology and Biostatistics UT Health San Antonio San Antonio Texas; ^4^ South Texas Veterans Health Care System San Antonio Texas; ^5^ Department of Pharmacology UT Health San Antonio San Antonio Texas; ^6^ Department of Cellular and Integrative Physiology UT Health San Antonio San Antonio Texas

**Keywords:** age‐specific mortality, gender differences, lifespan, sex differences, smoothed hazard estimation, somatotrophic axis

## Abstract

The female survival advantage is a robust characteristic of human longevity. However, underlying mechanisms are not understood, and rodent models exhibiting a female advantage are lacking. Here, we report that the genetically heterogeneous (UM‐HET3) mice used by the National Institute on Aging Interventions Testing Program (ITP) are such a model. Analysis of age‐specific survival of 3,690 control ITP mice revealed a female survival advantage paralleling that of humans. As in humans, the female advantage in mice was greatest in early adulthood, peaking around 350 days of age and diminishing progressively thereafter. This persistent finding was observed at three geographically distinct sites and in six separate cohorts over a 10‐year period. Because males weigh more than females and bodyweight is often inversely related to lifespan, we examined sex differences in the relationship between bodyweight and survival. Although present in both sexes, the inverse relationship between bodyweight and longevity was much stronger in males, indicating that male mortality is more influenced by bodyweight than is female mortality. In addition, male survival varied more across site and cohort than female survival, suggesting greater resistance of females to environmental modulators of survival. Notably, at 24 months the relationship between bodyweight and longevity shifted from negative to positive in both sexes, similar to the human condition in advanced age. These results indicate that the UM‐HET3 mouse models the human female survival advantage and provide evidence for greater resilience of females to modulators of survival.

## 1 INTRODUCTION

The female survival advantage is one of the most robust patterns in the study of human longevity (Austad & Bartke, 2015; Wisser & Vaupel, 2014). Women have a mortality advantage at almost every age in all developed countries (Gjonça, 1999). This advantage persists in middle‐ and lower‐income countries, disappearing only in a small number of countries where women face exceptional social disadvantages (World Health Organization, 2009). While this pattern of survival is well‐documented, the underlying biological mechanisms are unknown. Inbred strains of mice are overwhelmingly used as mammalian models for basic research, but their utility for studying sex differences in aging is limited by their susceptibility to strain‐specific diseases and their lack of a consistent female survival advantage (Austad, 2011; Austad & Bartke, 2015). The National Institute on Aging Interventions Testing Program (ITP) was designed to overcome strain‐specific peculiarities by using genetically heterogeneous UM‐HET3 mice, the result of a four‐way cross between [BALB/cJ × C57BL/6J]F1 mothers and [C3H/HeJ × DBA/2J]F1 fathers (Miller et al., 2007). The four‐way cross is used to generate populations of genetically diverse but related individuals in a reproducible manner (Roderick, 1963). The use of genetically heterogeneous mice reduces the effects of strain‐specific pathologies, because such mice show a broader representation of causes of death (Lipman, Galecki, Burke, & Miller, 2004). Female UM‐HET3 mice live longer than males as measured by median longevity, a result that has been observed consistently across multiple studies conducted by the ITP at all three of its study sites (Austad et al., 2016). However, a detailed study of the survival characteristics of each sex has not yet been conducted. The age‐specific mortality rate is the instantaneous hazard (the mortality at time *t*, given survival to *t*). This approach produces an estimate of mortality risk at each age across the lifespan. In contrast, the majority of animal longevity studies use averages or point estimates such as median and maximum lifespan to compare mortality between experimental groups. These commonly used approaches, however, cannot detect differences that can be uncovered by analysis of age‐specific mortality (Engelman, Seplaki, & Varadhan, 2017), such as sex differences unique to specific ages (Bronikowski, Morgan, Garland, & Carter, 2006), mortality convergence (decrease in relative differences in death rates between populations) (Jacobs, Cohen, Ein‐Mor, & Stessman, 2014), and reversal of mortality differences (Jatoi, Anderson, & Rosenberg, 2008). Most survival studies in mice and rats are not amenable to age‐specific mortality measurement, because their sample sizes are too small to obtain accurate estimates, particularly at early and late stages of life (Carey & Liedo, 1995).

The sample sizes of the studies of genetically heterogeneous mice in the Intervention Testing Program are large enough to obtain age‐specific mortality rates with enough statistical power to identify patterns of sex‐specific mortality unique to selected ages, as well as mortality rate convergence, divergence, and crossovers across the lifespan. Further, the large study populations enable identification of differences in bodyweight between males and females across the lifespan that could explain the sex difference in survival. Considerable evidence supports the idea that factors that control growth trajectory affect lifespan. Smaller animals within a species live longer, an observation found in rodents (Bartke, Heiman, Turyn, Dominici, & Kopchick, 2004) as well as other mammals (Greer, Canterberry, & Murphy, 2007). In UM‐HET3 mice, lifespan has been previously reported to be negatively correlated with bodyweight (Miller, Harper, Galecki, & Burke, 2002). Previous studies of bodyweight in the UM‐HET3 mice showed no effect of sex on the relationship between weight and lifespan (Harper, Galecki, Burke, & Miller, 2004; Miller et al., 2002). However, these prior analyses were conducted at a single site with significantly fewer mice than the current study (Miller et al., 2007). Here, we report findings from analyses of age‐specific mortality in male and female UM‐HET3 control (i.e., untreated) mice from the first six cohorts used to test a series of agents for their lifespan‐extending action. We also report analyses of the relationship between bodyweight and age‐specific mortality in the two sexes. The results not only indicate that the UM‐HET3 mouse is a useful model of the female human survival advantage, but also uncover evidence for greater resilience of females to modulators of survival.

## 2 RESULTS

### 2.1 Sex differences in overall survival, median lifespan, and maximum lifespan

**Figure 1:**
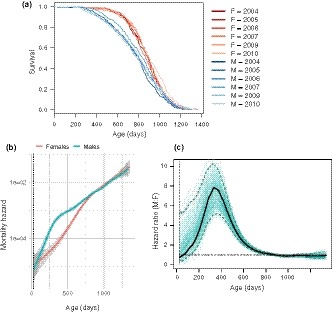
Sex‐specific survival dynamics of genetically heterogeneous UM‐HET3 mice. (a) Survival curves for male and female mice, showing the proportion surviving at each age. Survival curves are stratified by sex and study cohort (year). Survival for males (M) is shown in blue, females (F) in red. Average cohort size for males *n* = 332, females *n* = 282. Total number of animals *n* = 3,690. (b) Mortality hazard in male and female mice. Each line (males in blue, females in red) represents the smoothed estimated hazard as a function of age. Confidence intervals (95%) are shaded in gray. Vertical dotted line indicates age of weaning. (c) Ratio of male to female mortality hazard at each age in mice. Black line represents the hazard ratio of males to females as a function of age. Dashed blue lines demarcate the bootstrapped 95% confidence limits. Individual green lines represent hazard ratios estimated from resampling replicates. Horizontal dotted black line represents a hazard ratio of 1, indicating no difference in estimated hazard between males and females

Figure 1a shows the Kaplan–Meier survival curves of 3,690 control (i.e., pharmacologically untreated) mice pooled across all three sites (TJL = The Jackson Laboratory, UM = University of Michigan, UT = UT Health San Antonio), for six separate studies conducted over a 10‐year period. The survival curves are stratified by sex and study cohort. The effect of sex on survival (i.e., female survival exceeded male survival) was consistent throughout the six studies: Females had a longer median lifespan (887 days, 95% CI: 879–898) than males (803 days, 95% CI: 791–815) both when all the survival data were pooled and when the data were stratified by study cohort (Supporting information Table S1). Similarly, the effect of sex on overall survival was significant both in the pooled population and in five out of six study cohorts, as determined by log‐rank test (Supporting information Table S1). The effect of sex on maximum lifespan was determined by testing the difference in the proportion of mice in each sex that were long‐lived (defined as mice that survived until the age at which the total population, including both males and females, had reached 90% mortality). Using this measure, in contrast to median lifespan, there was no effect of sex on maximum lifespan in any study cohort (Survival at maximum lifespan in Females: 95% CI 0.079–0.121, Males: 95% CI 0.080–0.120).

### 2.2 Sex differences in age‐specific mortality

The presence of a sex difference in median lifespan and the absence of one for maximum lifespan indicate that the sex difference in mortality varies with age. To delineate the nature of this variation, we calculated the effect of sex on age‐specific mortality across the lifespan. We estimated age‐specific mortality by computing the smoothed hazard for each sex using data pooled across all cohort years. Figure 1b shows estimated mortality hazard with 95% confidence intervals, stratified by sex: The hazard in males is greater than that of females in the first 600 days of age, after which there is a convergence in mortality between the sexes. Figure 1c shows the marked variation in the hazard ratio of males to females as a function of age. The hazard ratio, indicating the relative difference in male and female mortality rates, rises sharply from early adulthood to a peak around 350 days and steadily declines thereafter until about 800 days, when male and female mortality rates become indistinguishable. This pattern of greatest mortality difference followed by convergence of mortality rates between males and females is present both in the pooled population from all study cohorts and within each of the six study cohorts (Supporting information Figure S4).

### 2.3 Site‐specific variation in the sex differential of mortality

**Figure 2:**
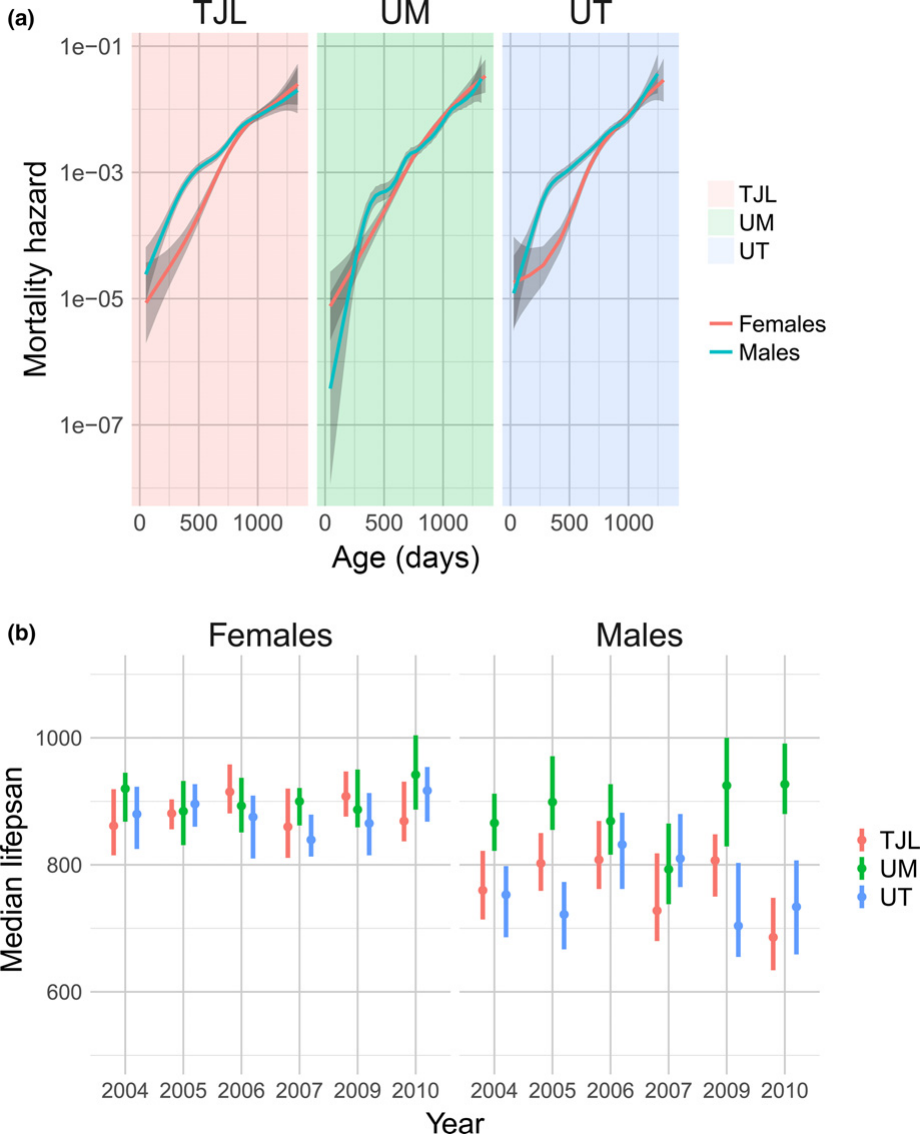
Site differences in survival across study cohorts, showing greater variability in males. Mortality hazard in male and female mice is shown for each site (a). Each line (males in blue, females in red) represents the smoothed estimated hazard as a function of age. Confidence intervals (95%) are shaded in gray. Lower panel (b) shows median lifespan of males and females at each site, split by study cohort (year). TJL = The Jackson Laboratory (red), UM = University of Michigan (green), UT = UT Health San Antonio (blue)

Previous reports have noted that median lifespans of males at UM are longer than those of males at TJL and UT (Harrison et al., 2009; Miller et al., 2007). To further investigate this site‐specific variability, we compared the survival characteristics between sites across cohort years. While UT and TJL maintained a persistent sex difference both in the pooled dataset (including all cohorts) and within each individual cohort year, we found no difference in median lifespan or overall survival between males and females at UM (Table S2). In order to determine whether the effect of site was localized to survival in specific age ranges, we computed the age‐specific hazard at each site (Figure 2a). Indeed, males at the UM site appeared to have a lower mortality rate than males at the two other sites around 500 days of age. Notably, by comparing estimates of the sex‐specific mortality hazards at all ages, in contrast to our results using median and overall survival, we detected a significant difference between male and female survival, even at the UM site. The female survival advantage was reduced but maintained at UM at the age identified in our pooled initial analyses as the age of peak mortality difference between the sexes (Figure 1c).

The diminished sex difference in survival at UM suggested an interaction between site and sex on survival. In order to quantify this effect, we performed a Cox regression of survival time upon sex, site, and sex–site interaction variables in the combined population across all cohort years. We found a significant interaction between sex and site: While there was no difference between females at each site, there was a significant interaction effect of sex and site, supporting a difference between males at UM compared to males at UT and TJL (HR = 0.661, *p* < 0.001). We obtained similar results when we repeated our Cox regression in each cohort year: When we stratified by both sex and study cohort to determine the effect of site on survival in each subgroup, we observed a pronounced effect of site on lifespan in the male mice but not females. Supporting information Figure S5 shows the coefficients of the Cox regression for each site: There was a pronounced effect of site on lifespan in the male mice. The diminished sex difference in mortality at UM is due to the decreased mortality in males compared to males at UT and TJL in a majority of cohorts. In contrast, there was no effect of site on survival in female mice in any study cohort (Supporting information Figure S5). The extraordinary stability of female lifespan is reflected in a markedly lower variance across different sites and cohort years (Figure 2b).

### 2.4 Sex‐specific differences in the relationship between bodyweight and lifespan

**Figure 3:**
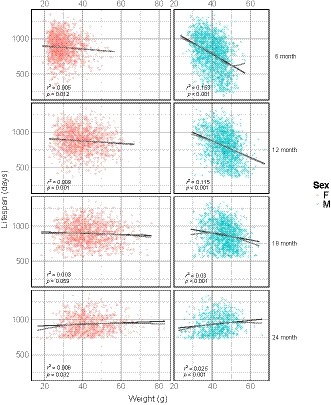
Relationship between bodyweight and lifespan by sex and age. Each panel shows the scatterplot and linear regression line of lifespan by bodyweight in males (blue) and females (red) at four different ages of bodyweight measurement (age in months, righthand axis). A nonlinear fit is shown in gray to assess departure from linearity

One potential basis for the sex differences in age‐specific mortality rates is variation in bodyweight. Lower bodyweights and smaller body sizes have previously been correlated with increased lifespan (Bartke et al., 2004). Figure 3 shows the effect of sex on the relationship between bodyweight and lifespan for each age of bodyweight measurement. We performed a regression of lifespan on bodyweight at each measurement age (6, 12, 18, and 24 months) and found a significant correlation between bodyweight and lifespan at all ages (Table 1). There was little observed departure from linearity. Table 2 shows the results of a similar regression of lifespan on bodyweight, accounting for sex. In males, there was a negative correlation between bodyweight and lifespan using bodyweights measured at 6, 12, and 18 months of age (*β* = −15.72, −11.75, −4.89 days/g, respectively, *p* < 0.001) (Figure 3 and Table 2). The relationship between weight and lifespan in males changed with age, such that the negative correlation between bodyweight and lifespan observed became weaker at each successive age of measurement, eventually reversing at 24 months of age (*β* = 2.99 days/g, *p* < 0.001). In contrast, the effect of weight on lifespan in females was much smaller or not significant at each age of bodyweight measurement (*β* = −2.16 days/g, −1.87, and 1.02 at 6, 12, and 24 months, respectively, *p* < 0.05; n.s. at 18 months). Indeed, when sex and a sex–weight interaction term were added to the model, we found a significant interaction between sex and weight (Supporting information Table S3).

**Table 1:** Results of linear regression of lifespan on bodyweight (stratified by age of bodyweight measurement)

Age of measurement (months)	Variable	Coefficient	*SE*	*p*‐value	*R* ^2^	Standardized coefficient
6	Weight	−9.40	0.504	<0.001*	0.115	−0.339
12	Weight	−6.39	0.476	<0.001*	0.0693	−0.263
18	Weight	−2.15	0.403	<0.001*	0.0120	−0.110
24	Weight	1.62	0.406	<0.001*	0.0115	0.107

* *p*‐value < 0.05.

**Table 2:** Results of linear regression of lifespan on sex and bodyweight (stratified by age of bodyweight measurement)

Sex	Age of measurement (months)	Variable	Coefficient	*SE*	*p*‐value	Standardized coefficient
Females	6	Weight	−2.16	0.982	0.028*	−0.078
	12	Weight	−1.87	0.641	0.004*	−0.077
	18	Weight	−0.85	0.477	0.075	−0.043
	24	Weight	1.02	0.487	0.037*	0.067
Males	6	Weight	−15.72	0.886	<0.001*	−0.567
	12	Weight	−11.75	0.849	<0.001*	−0.484
	18	Weight	−4.89	0.779	<0.001*	−0.249
	24	Weight	2.99	0.729	<0.001*	0.197

* *p*‐value < 0.05.

### 2.5 Effect of site on the relationship between bodyweight and lifespan in males

We further addressed the question of whether the environmental effect of site interacts with bodyweight to explain the greater survival of males at UM compared to those at UT and TJL. Given that the bodyweight showed the strongest correlation with lifespan when measured at 6 months, we performed a regression of lifespan on bodyweight measured at 6 months and tested the significance of the interaction between site and bodyweight. Once we controlled for the effect of bodyweight, the effect of site on lifespan was no longer significant (Supporting information Table S5). We found no significant effect of the interaction between weight and site on lifespan, suggesting that the relationship between weight and lifespan is consistent across sites (Supporting information Table S4).

## 3 DISCUSSION

### 3.1 Sex difference in early‐ and middle‐age mortality

A major revelation of this analysis is that the previously noted female survival advantage observed in genetically heterogeneous UM‐HET3 mice (Austad et al., 2016; Miller et al., 2007) is mainly the result of sex differences in early‐ and mid‐adult mortality. The mortality difference is greatest in the first half of life, peaking around 350 days of age and decreasing steadily thereafter until reaching convergence around the median lifespan. This finding underscores the importance of age‐specific survival analysis, which can reveal nonuniformity in the mortality rate across the lifespan and, as a result, indicate that the causes of mortality, whether genetic, pathological, or environmental, are age‐specific (Jatoi et al., 2008). Thus, studies to uncover the underlying mechanisms of aging and mortality for this strain, and likely for other animal models, will necessitate treating age as an independent variable (Watson & Leadbetter, 1964).

The observation that the sex differential in mortality of UM‐HET3 mice is most prominent in early adulthood and middle age was not apparent in previous publications, because the analyses were not designed to identify age‐specific trends. In the aging literature, longevity is often described using the Gompertz model in comparative studies (Finch, Pike, & Witten, 1990) or Kaplan–Meier estimator and mean/median survival in experimental studies (Yang et al., 2011). These approaches, although useful, have limitations (Hagar & Dukic, 2015). Violations of the Gompertzian assumption of constant mortality acceleration are not uncommon in observed populations, which has led to the development of extensions to the Gompertz model and piecewise models of mortality rate (Economos, 1979). Kaplan–Meier survival analysis is subject to its own set of limitations (Li, Han, Hou, Chen, & Chen, 2015). This is exemplified by our result for study cohort 2009, wherein male and female Kaplan–Meier survival curves cross at 1,052 days of age (Supporting information Figure S7): No significant difference in overall survival between males and females was found (Supporting information Table S1). In contrast, when comparing estimates of the hazard function across all ages, we find that cohort 2009 is consistent with the other years in that there is a significant difference between mortality hazard in males and females in the first half of life between 250 and 750 days of age.

In recent decades, smoothed nonparametric estimation of hazard rates has become a common statistical tool for analysis of censored survival data (Müller & Wang, 1994). Although some of these techniques have been published, their applicability to studies of rodent aging is limited, because they require larger sample sizes than customary in experimental rodent aging research (Carey & Liedo, 1995; Pletcher, Khazaeli, & Curtsinger, 2000). Moreover, the development and widespread availability of statistical software used for smoothing procedures were limited before the development of open‐source implementations (Wang, 2005). The availability of open‐source implementations for smooth nonparametric estimation of the hazard function (Hagar & Dukic, 2015; Rebora, Salim, & Reilly, 2014) and larger datasets such as the ITP enabled us to describe age‐specific characteristics of rodent mortality with higher resolution than the Gompertz model (Gavrilova & Gavrilov, 2015) and with less variance than a point estimator such as the Kaplan–Meier (Wang, 1988).

Our findings in mice parallel in humans both the early adulthood peak in higher male mortality risk (Supporting information Figure S6) and the subsequent convergence of male and female mortality rates in the oldest old (Jacobs et al., 2014). Mortality rate convergence in late life is a well‐documented phenomenon that is observed in human populations across nations with widely varying life expectancies (Edwards & Tuljapurkar, 2005; Engelman et al., 2017; Jacobs et al., 2014). The biological basis for the sex difference in mortality in early adulthood and beyond, which has been observed not only in humans but also in other primates (Bronikowski et al., 2011), remains poorly understood. Although sex differences in behavior are associated with and likely contribute to the greater mortality rate in males (Beeman, 1947; Wingard, 1982), the female survival advantage is consistent across widely diverse populations (Austad & Bartke, 2015) and remains even after causes of mortality associated with sexual dimorphic behavior (e.g., violence or motor vehicle accidents) are removed (Carnes & Olshansky, 1997). Similarly, the female survival advantage is observed in primate populations both in the wild and in the captivity, suggesting that the difference is not solely due to the costs of sex‐specific behaviors (e.g., dispersal, exposure to predation, or intrasexual competition; Bronikowski et al., 2011). In our study, mice were maintained under conditions in which mortality related to aggression was greatly minimized. In the event of significant injury due to fighting, which occurred in approximately 6% of cages housing males (Supporting information Figure S3), all mice in the cage were censored (Miller et al., 2007). Given that mortality due to fight wounds was minimized, the striking difference in mortality rates between males and females we observed in early‐ and mid‐adult life is likely driven at least in part by other biological mechanisms. One approach to determining these mechanisms is to identify compounds that have differential effects on lifespan between males and females, in particular, drugs that selectively reduce the early adulthood excess mortality of males (Austad & Bartke, 2015; Austad et al., 2016). Indeed, most interventions in the ITP program have sex‐specific effects on mortality (Harrison et al., 2014). Identification of separate mechanisms that can be localized to specific age ranges responsible for mortality could provide an understanding of the biological underpinnings of sex‐specific survival dynamics.

### 3.2 Sex difference in relationship between lifespan and bodyweight

The second sex difference we report in this study is in the relationship between lifespan and bodyweight. An inverse relationship between bodyweight and lifespan has been widely observed in rodents (Bartke et al., 2004), other mammals (Greer et al., 2007), and humans (Samaras, Elrick, & Storms, 2003). Our results confirm these observations and extend them by being among the first to identify a sex difference in the relationship between bodyweight and lifespan in mammals (Norry & Loeschcke, 2002; Warkentin, Espie, Lieske, & James, 2016): namely, that this relationship is much stronger in males than in females. This sexual dimorphism is not the trivial outcome of the disproportionate number of heavy males. While UM‐HET3 males are heavier than females (Supporting information Table S6), exclusion of these heavy animals from the dataset does not meaningfully change the results of our analysis (Supporting information Tables S7–S8). Further, the relationship between bodyweight and life expectancy varies with age. The strength of the correlation, as measured by the correlation coefficient, declines progressively from 6 to 18 months, confirming previous findings (Miller et al., 2002). Of note, we further find that the negative correlation between bodyweight and lifespan is inverted at later ages. This parallels the clinical pattern in humans, where the relative risk of death associated with greater bodyweight is lower for older than for younger adults (Stevens et al., 1998).

A potential mechanism underlying this sex‐dependent bodyweight–lifespan correlation is the activity of the somatotrophic axis. Whether the relationship between lifespan and IGF‐1 levels is sexually dimorphic remains to be determined. Prior results in the UM‐HET3 mice support the hypothesis that greater somatotrophic activity, as measured by circulating IGF‐1 levels, contributes to shorter lifespan in males but not females (Harper, Wolf, Galecki, Pinkosky, & Miller, 2003). Other studies report an inverse relationship between IGF‐1 levels and lifespan in both sexes across inbred mouse strains (Yuan et al., 2009).

### 3.3 Sex‐specific site effect on lifespan

The third sex difference revealed in this study is the greater variance in male compared to female survival across site and study year. This unanticipated finding suggests that males are more sensitive than females to environmental factors that influence survival. Prior studies across vertebrate species have shown that males experience greater increase in mortality in response to environmental stress than females (Dunham, Maitner, Razafindratsima, Simmons, & Roy, 2013). The corollary of the greater sensitivity of males to mortality associated with intrinsic (e.g., bodyweight‐related) and environmental stresses is that females are more resistant to life‐threatening perturbations. This idea is supported in humans by the well‐known mortality–morbidity paradox wherein women show greater survival in spite of a higher apparent disease burden (Kulminski et al., 2008). It remains to be seen whether the higher mortality rate in males is the product of the action of testicular androgens or, conversely, if the presence of estrogens in females contributes to their lower mortality, or if a combination of both factors is involved (Cheng & Nelson, 2018). Moreover, hormonal underpinnings can either result from direct action of the steroids during adulthood (i.e., activational effects), or developmental programming (i.e., organizational effects) during fetal or postnatal life (Arnold, 2014). Nonhormonal, extra‐gonadal factors driven by differences in gene expression emanating from sexual dimorphism of the sex chromosomal complement may also play a role (Arnold, 2014).

In conclusion, the UM‐HET3 mouse provides for the first time a useful murine model to probe the basis for sex differences in age‐specific survival and resistance to perturbations influencing survival. One caveat to this model is that the magnitude of the sex differential can vary markedly across study sites. The power to detect sex differences in survival and, by inference, their underlying causes, varies by study site, and consideration must be given to this observation in the design of future studies. One of the great strengths of the ITP has been its attention to replication across three study sites. Had the ITP only been conducted in one of the three sites, the power might have been insufficient to detect the observations of this paper. Altogether, these results add to the understanding of the UM‐HET3 mouse, a model increasingly used for the study of aging and lifespan‐extending interventions, and underscore its potential for interrogating the basis for sex differences in aging that bear striking resemblance to those of humans.

## 4 EXPERIMENTAL PROCEDURES

### 4.1 Mouse survival dataset

Data were obtained from the Interventions Testing Program, a large‐scale screening program for the evaluation of candidate agents with the potential to delay aging (Miller et al., 2007). Key features of the ITP include the use of genetically heterogeneous mice and simultaneous replication at three test sites: The Jackson Laboratory (TJL), University of Michigan (UM), and UT Health San Antonio (UT). All mice used in these studies were of the UM‐HET3 stock, a four‐way cross between [BALB/cJ × C57BL/6J]F1 mothers and [C3H/HeJ × DBA/2J]F1 fathers. Survival data were obtained from six longevity studies conducted between the years of 2004 and 2013. Each study cohort is defined by start year (2004, 2005, 2006, 2007, 2009, and 2010), during which all animals at all sites are bred and enrolled in the lifespan study. Animals enrolled in ITP studies were monitored daily from the age of weaning until the end of their natural lifespan, defined as the age of death or euthanasia following signs of severe moribundity (Miller et al., 2007). Data analyzed in this study were from a total of 3,690 animals in the control groups at all three sites. Raw mortality data for the ITP mice can be obtained by request to Dr. Richard A. Miller (millerr@umich.edu).

### 4.2 Animal care and maintenance

UM‐HET3 mice were bred at each of the three test sites from CByB6F1/J (JAX stock #100,009) mothers and C3D2F1/J (JAX stock #100,004) fathers. The first litter from each breeding cage was discarded to avoid enrolling mice born to primiparous dams, which may receive inferior nutrition compared to offspring of subsequent pregnancies. Mice were weaned into same‐sex cages (three males or four females per cage) at 19–21 days of age. Mice were housed in plastic cages with metal tops and corn‐cob bedding. Bedding and diet were obtained from the same supplier at each site. Temperature was maintained within the range of 21–23°C. Other environmental conditions (e.g., light/dark cycle) were coordinated between the sites. Each mouse colony was evaluated for the presence of pathogens four times each year. Detailed methods can be found in prior publications by the ITP (Miller et al., 2007).

### 4.3 Analysis

Sex‐specific survival analysis: The effect of sex on survival was evaluated using several measures: by comparing median lifespan, maximum lifespan, and the general survival function. The frequency and distribution of censored animals are shown in Supporting information Figures S1–S3. The ability of each sex to reach extreme longevity was evaluated as previously described (Wang, Li, Redden, Weindruch, & Allison, 2004). Briefly, the maximum lifespan was approximated as the age by which the pooled population (including both males and females) has reached 90% mortality. Then, within each sex, the proportion of mice still alive at this age was calculated. The effect of sex on maximum lifespan was then evaluated by comparing the proportion in males to the proportion in females that reach maximum lifespan using a Fisher exact test. The Kaplan–Meier survival curves were estimated for each sex. The difference between the male and female survival curves was tested using log‐rank test using the *survival* package (Therneau, 2015). Confidence intervals for the age of median survival were calculated based on Aalen estimator of the hazard variance (Therneau, 2015). Age‐specific mortality, the instantaneous rate of mortality at each age, was determined with a piecewise polynomial B‐spline hazard model assuming a Poisson distribution (Lambert & Eilers, 2005; Rebora et al., 2014) using the *bshazard* package (Rebora et al., 2014). A nonparametric smoothed estimate of baseline hazard rate was obtained for each sex using survival data pooled across all study cohorts. The confidence intervals for hazard rate were estimated using 1,000 bootstrapped replications.

Site‐specific survival analysis: To determine the effect of sex and study site on survival, a multivariate Cox proportional hazards model was fitted. Sex, study site, and the interaction between sex and study site were included as variables in the Cox regression. The analysis was repeated for both the full dataset and datasets stratified by study year. Significance of the effect of each variable was assessed by likelihood ratio test.

Bodyweight analysis: Bodyweight data were obtained for 2,946 mice (1,352 females, 1594 males). Weight measurements were collected at 6, 12, 18, and 24 months of age. We excluded 56 females and 93 males from bodyweight analysis that were censored in the course of the longevity studies. Out of the remaining 8,844 weight measurements, eight weight measurements (~0.1%) were identified as erroneous (the recorded date of measurement was after the recorded date of death) and excluded from our analysis. We conducted a multiple regression to predict lifespan based on the following variables: weight, sex, and the interaction between sex and weight. Since bodyweight was measured at multiple time points, we stratified the analysis by the age at which the weight was recorded. We further regressed lifespan on bodyweight, study site, and bodyweight–site interaction variables.

All analyses were performed in the R environment for statistical computing 3.3.0 (R Core Team, 2016) within an accountable data analysis process (Gelfond, Goros, Hernandez, & Bokov, 2018).

## CONFLICT OF INTEREST

None declared.

## AUTHOR CONTRIBUTIONS

CJC, JALG, and JFN designed the study; CJC performed analysis under the direction of JALG and JFN. CJC wrote the manuscript with input, contributions, and comments and critical revisions from all authors.
