# Supplementary material for: Genetically heterogeneous mice exhibit a female survival advantage that is age‐ and site‐specific: Results from a large multi‐site study
Source: Aging Cell. 2019 Feb 23;18(3):e12905. doi: 10.1111/acel.12905 (PMC6516160; doi:10.1111/acel.12905)
Supplement: Supplementary file 1 [file ACEL-18-e12905-s001.docx]

**Supplemental Tables**

Table S1. Median lifespan and results of log-rank test of sex difference in overall survival in each cohort year (pooled across all sites)

| *Year* | *Median Lifespan (days) in Females (95% CI)* | *Median Lifespan (days) in Males (95% CI)* | *Significance (log-rank test)* |
| --- | --- | --- | --- |
| 2004* | 892 (860 – 916) | 790 (765 – 819) | 3.74e-05^†^ |
| 2005* | 887 (862 – 905) | 797 (769 – 835) | 4.15e-03^†^ |
| 2006* | 891 (873 – 911) | 834 (800 – 869) | 4.22e-03^†^ |
| 2007* | 866 (835 – 892) | 786 (749 – 827) | 2.07e-03^†^ |
| 2009* | 891 (869 – 915) | 807 (782 – 841) | 8.57e-02 |
| 2010* | 902 (873 – 940) | 784 (739 – 819) | 7.96e-05^†^ |
| All years combined* | 887 (879 – 898) | 803 (791 – 815) | 1.65e-14^†^ |

*Significant difference in median lifespan between sexes

^†^Significant difference in overall survival between sexes

Table S2. Median lifespan and results of log-rank test of sex difference in overall survival at each site (pooled across cohort years)

| *Site* | *Median Lifespan (days) in Females (95% CI)* | *Median Lifespan (days) in Males (95% CI)* | *Significance (log-rank test)* |
| --- | --- | --- | --- |
| TJL* | 886 (869-902) | 779 (751-797) | 6.46e-13^†^ |
| UM | 901 (887-918) | 874 (856-903) | 0.963 |
| UT* | 876 (859-896) | 760 (729-782) | 3.20e-12^†^ |

*Significant difference in median lifespan between sexes

^†^Significant difference in overall survival between sexes

Table S3 Results of multiple linear regression of lifespan on sex, weight, and sex-weight interaction (stratified by date of bodyweight measurement)

| **Age of Measurement** | **Variable** | **Coefficient** | **SE** | **P-value** | **Standardized Coefficient** |
| --- | --- | --- | --- | --- | --- |
| 6 months (R^2^ = 0.148) | |  |  |  |  |
|  | **Sex** (main effect) | |  |  |  |
|  | Female^1^ | 0 | - | - | - |
|  | Male | -84.1 | 13.5 | <0.001* | -0.195 |
|  | **Weight** (slope) | |  |  |  |
|  | Female^1^ | -2.16 | 0.982 | 0.028* | -0.078 |
|  | Male | -13.6 | 1.32 | <0.001* | -0.489 |
| 12 months (R^2^ = 0.108) | |  |  |  |  |
|  | **Sex** (main effect) | |  |  |  |
|  | Female^1^ | 0 | - | - | - |
|  | Male | -9.91 | 8.64 | 0.252 | -0.026 |
|  | **Weight** (slope) | |  |  |  |
|  | Female^1^ | -1.87 | 0.641 | 0.004* | -0.077 |
|  | Male | -9.88 | 1.06 | <0.001* | -0.407 |
| 18 months (R^2^ = 0.024) | |  |  |  |  |
|  | **Sex** (main effect) | |  |  |  |
|  | Female^1^ | 0 | - | - | - |
|  | Male | -1.34 | 7.85 | 0.865 | -0.004 |
|  | **Weight** (slope) | | | | |
|  | Female^1^ | -0.85 | 0.477 | 0.074 | -0.043 |
|  | Male | -4.04 | 0.913 | <0.001* | -0.206 |
| 24 months (R^2^ = 0.015) | |  |  |  |  |
|  | **Sex** (main effect) | |  |  |  |
|  | Female^1^ | 0 | - | - | - |
|  | Male | -0.31 | 7.16 | 0.965 | -0.001 |
|  | **Weight** (slope) | |  |  |  |
|  | Female^1^ | 1.02 | 0.487 | 0.037* | 0.067 |
|  | Male | 1.97 | 0.877 | 0.025* | 0.130 |

^1^Reference category

*p<0.05

### Table S4 Results of multiple linear regression of lifespan on weight, site, and weight-site interaction (stratified by sex)

| **Sex** | **Variable** | **Coefficient** | **SE** | **P-value** | **Standardized Coefficient** |
| --- | --- | --- | --- | --- | --- |
| *Females* (R^2^ = 0.0077) | | | | | |
|  | **Site** (main effect) | |  |  |  |
|  | TJL^1^ | 0 | - | - | - |
|  | UM | 62.0 | 35.9 | 0.084 | 0.288 |
|  | UT | 0.04 | 22.9 | 0.999 | 0.001 |
|  | **Weight** (slope) |  |  |  |  |
|  | TJL^1^ | -2.52 | 1.43 | 0.0793 | -0.084 |
|  | UM | 3.98 | 2.64 | 0.132 | 0.019 |
|  | UT | -0.12 | 1.99 | 0.953 | -0.004 |
| *Males* (R^2^ = 0.1550) | | | | | |
|  | **Site** (main effect) | |  |  |  |
|  | TJL^1^ | 0 | - | - | - |
|  | UM | -2.35 | 19.33 | 0.903 | -0.008 |
|  | UT | -20.40 | 14.80 | 0.168 | -0.508 |
|  | **Weight** (slope) |  |  |  |  |
|  | TJL^1^ | -15.40 | 2.05 | <0.001* | -0.384 |
|  | UM | 0.38 | 3.07 | 0.902 | 0.001 |
|  | UT | -0.47 | 2.76 | 0.864 | -0.012 |

^1^Reference category

*p<0.05

Note: Regression analyses were performed using bodyweight measurements recorded at 6 months of age.

Table S5 Results of multiple linear regression of lifespan on weight and site (stratified by sex)

| **Sex** | **Variable** | **Coefficient** | **SE** | **P-value** | **Standardized Coefficient** |
| --- | --- | --- | --- | --- | --- |
| *Females* (R^2^ = 0.006) | | | | | |
|  | **Site** (main effect) |  |  |  |  |
|  | TJL^1^ | 0 | - | - | - |
|  | UM | 9.37 | 12.5 | 0.452 | 0.044 |
|  | UT | 1.01 | 12.2 | 0.934 | 0.033 |
|  | **Weight** (slope) |  |  |  |  |
|  | All sites | -1.91 | 0.91 | 0.036* | -0.063 |
| *Males* (R^2^ = 0.155) | | | | | |
|  | **Site** (main effect) |  |  |  |  |
|  | TJL^1^ | 0 | - | - | - |
|  | UM | -5.10 | 16.5 | 0.757 | -0.017 |
|  | UT | -21.0 | 14.2 | 0.140 | -0.523 |
|  | **Weight** (slope) |  |  |  |  |
|  | All sites | -15.5 | 1.18 | <0.001* | -0.386 |

^1^Reference category

*p<0.05

Regression analyses were performed using bodyweight measurements recorded at 6 months of age.

Table S6 Bodyweight distribution of animals in ITP

| **Weights measured at 6 months** | | |
| --- | --- | --- |
| **Weight group (g)** | **Females** | **Males** |
| (0,10] | 0 | 0 |
| (10,20] | 8 | 0 |
| (20,30] | 766 | 48 |
| (30,40] | 401 | 652 |
| (40,50] | 69 | 684 |
| (50, max] | 7 | 51 |

Table S7 Results of linear regression of lifespan on bodyweight, heaviest bodyweights removed

(All animals weighing more than 50 grams excluded)

| **Variable** | **Coefficient** | **SE** | **P-value** | **Standardized Coefficient** |
| --- | --- | --- | --- | --- |
| Weight (Female) | -2.03 | 1.03 | 0.047 | -0.07 |
| Weight (Male) | -16.8 | 0.97 | <0.001 | -0.51 |

*p<0.05

Regression analyses were performed using bodyweight measurements recorded at 6 months of age.

Table S8 Results of linear regression of lifespan on bodyweight, within comparable weight group

(Including only weights between 30-40 grams, the weight category with greatest overlap between males and females)

| **Variable** | **Coefficient** | **SE** | **P Value** | **Standardized Coefficient** |
| --- | --- | --- | --- | --- |
| Weight (Female) | 2.93 | 3.79 | 0.439 | -0.041 |
| Weight (Male) | -16.2 | 2.89 | <0.001 | -0.184 |

*p<0.05

Regression analyses were performed using bodyweight measurements recorded at 6 months of age.

**Supplemental figures**

**Figure S1. Censoring times in the ITP mortality dataset.** Histogram depicting the frequency distribution of censoring and mortality events by age. Events from all sites, years, and sexes are included.

**Figure S2. Censoring times for female mice at each site** Histograms depicting the frequency distribution of censoring and mortality events by age in female mice. Each panel shows the distribution at a single site for a single cohort year (TJL = The Jackson Laboratory (red), UM = University of Michigan (green), UT = UT Health San Antonio (blue)).

**Figure S3. Censoring times for male mice at each site.** Histograms depicting the frequency distribution of censoring and mortality events by age in male mice. Each panel shows the distribution at a single site for a single cohort year (TJL = The Jackson Laboratory (red), UM = University of Michigan (green), UT = UT Health San Antonio (blue)). ****

**Figure S4. Mortality hazard in male and female mice by study cohort.** Each panel shows the smoothed estimated hazard as a function of age in mice from a single study cohort (year). Confidence intervals (95%) are shaded in grey. Dotted line indicates age of weaning.

**Figure S5. Effect of site on survival in males and females, split by study cohort (year).** Hazard ratio (estimated from Cox proportional hazards model) for the effect of each study site shown with confidence intervals. TJL = The Jackson Laboratory (red), UM = University of Michigan (green), UT = UT Health San Antonio (blue).

**Figure S6. Sex difference in mortality in human populations.** Death rates at each age in men (blue) and women (red). Data from Human Mortality Database (https://www.mortality.org/) for the United States (2000-2009). Dotted line represents the average age of puberty (12.34 years).
