# Supplementary material for: Genetically heterogeneous mice exhibit a female survival advantage that is age‐ and site‐specific: Results from a large multi‐site study
Source: Aging Cell. 2019 Feb 23;18(3):e12905. doi: 10.1111/acel.12905 (PMC6516160; doi:10.1111/acel.12905)
Supplement: Supplementary file 2 [file ACEL-18-e12905-s002.pdf]

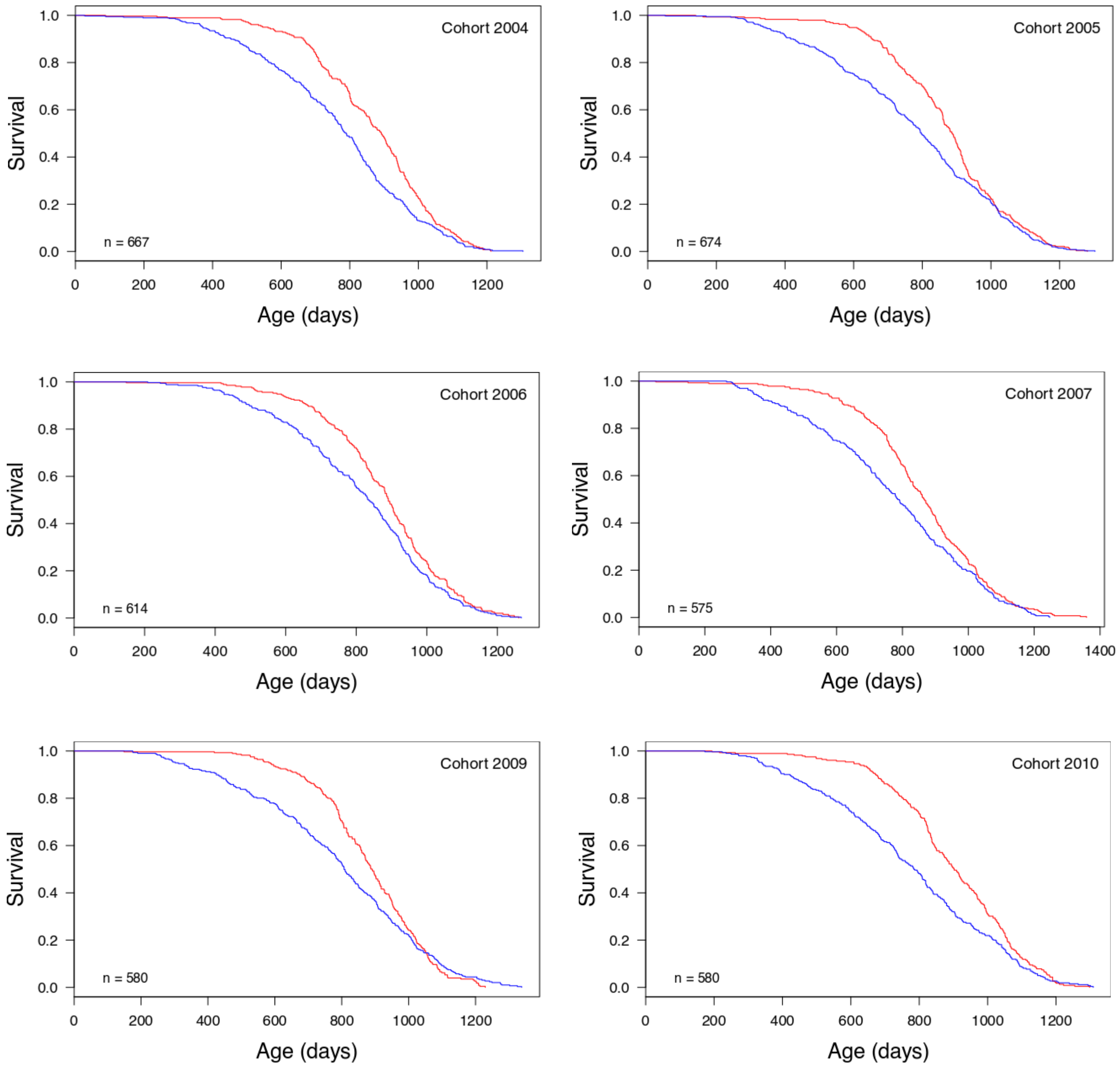

Figure S7. Survival curves for male and female mice by study cohort.
Each panel contains two survival curves showing the proportion surviving at each age in males and females in a specific study cohort. Survival for males (M) is shown in blue, females (F) in red.
